# Predictors for tuberculosis co-infection in people living with HIV/AIDs

**DOI:** 10.4314/ahs.v21i3.6

**Published:** 2021-09

**Authors:** Esra Zerdali, İnci Yılmaz Nakir, Serkan Sürme, Uğurcan Sayılı, Mustafa Yıldırım

**Affiliations:** 1 Haseki Education Research Hospital, Infectious Diseases and Clinical Microbiology, İstanbul, Turkey; 2 Karaköprü District Health Directorate, Public Health, Şanlıurfa, Turkey

**Keywords:** Tuberculosis, HIV viral load, CD4 cell counts

## Abstract

**Background/aim:**

Tuberculosis (TB) is one of the most common chronic infectious conditions causing mortality and severe outcomes, particularly in people living with HIV/AIDS (PLWHA). In this study, we aimed to determine the prevalence and predictors of TB among PLWHA.

**Materials and Methods:**

We conducted a retrospective and single-center study of adults (≥18 years) PLWHA registered at our tertiary teaching and research hospital between 2000 and 2016.

**Results:**

A total of 711 PLWHA were included. Of whom, 633 (89.0%) were male. Mean age was 36.53 ±11.55 years (range, 17–79). Thirty-eight (5.3%) patients were diagnosed with active TB. TB development was associated with low CD4+ lymphocyte count (p<0.001), high viral load (p=0.040) and alcohol consumption (p=0.004) but no association with age (p=0.392), gender (p=0.928) and duration since anti-retroviral therapy initiation (p=0.788) was found. Also, a receiver operating characteristic analysis showed that the area under the curves of CD4+ lymphocyte count as a predictor for TB development in PLWHA was 0.717 (p<0.001).

**Conclusion:**

There are still clinical challenges to predict TB diagnosis. However, CD4+ lymphocyte count and viral load may be considered as valuable predictors for TB development. Also, community strategies to reduce harmful effect of alcohol use should be developed.

## Introduction

Tuberculosis (TB) is one of the most common chronic infectious conditions causing mortality and severe outcomes, particularly in people living with HIV/AIDS (PLWHA). Additionally, it causes a significant increase in health expenditures due to prolonged hospitalization. Guidelines recommend HIV testing and counseling to all patients with TB. Therefore, patients with TB warrant prompt initiation of antituberculous therapy and HIV testing. In TB patients co-infected with HIV/AIDS, antiretroviral therapy (ART) is also necessary regardless of CD4+ T lymphocyte count^1^,^2^.

The decision to initiate treatment for TB is based on patient (age, risk for progression, exposure risk) and public health factors (concern for loss to follow-up, high transmission risk) as well as clinical (life-threatening conditions and typical symptoms), laboratory and radiographic findings. Also, empiric treatment should be initiated immediately even before the test results in patients who have a high possibility of having TB1.

HIV infection is one of the greatest risk factors for the development of TB which is associated with increased HIV replication and enhanced cytokine expression^3^. Therefore, it is important to determine the prevalence and associated risk factors of TB among PLWHA. In this study, we aimed to assess the prevalence and predictors of TB/HIV coexistence.

## Materials and Methods

We conducted a retrospective and single-center study of adults (≥18 years) PLWHA registered at our tertiary teaching and research hospital between 2000 and 2016. The patients followed up for at least 2 years were included in the study. PLWHA who had no proper follow-up datasheet or missing major data were excluded. The demographic data including age, gender, sexual orientation, and the localization of TB (pulmonary vs. extrapulmonary) and laboratory test results including baseline CD4+ T cell count, HIV RNA and tuberculin skin test (TST) were collected and assessed retrospectively from follow-up datasheets.

The diagnosis of TB was made basis on a combination of the following; the history of fever longer than onemonth duration, cough longer than three weeks duration, significant weight loss, contact with an adult with confirmed TB, and presence of acid-fast bacilli (AFB) in sputum/body fluids, abnormal radiological results, positive TST, and nonresponse to conventional antibiotics^1^.

Definite case of TB was defined as a patient with *Mycobacterium tuberculosis* complex identified from a clinical specimen by culture or polymerase chain reaction. A case of TB was defined as definite case of TB or one in which a clinician has diagnosed TB and has decided to treat the patient with a full course of TB treatment. Pulmonary TB was defined as a case of TB involving the lung parenchyma. Extrapulmonary TB was defined as a case of TB involving organs other than the lungs. TB pleural effusion, without radiographic abnormalities in the lungs, was also defined as a case of extrapulmonary TB.

The cases were divided into two groups according to TB diagnosis, and comparative analyses were applied. For the evaluation of the TST performed during TB screening, a diameter of 5 or more millimeters in the indurated area was considered positive^4^. Mortality was defined as all-cause mortality during TB treatment. Additionally, all-cause mortality during a 20-year study period (between 2000–2020) was evaluated. Mortality was recorded via identity numbers of the patients using the National Death Certificate Database (https://obs.saglik.gov.tr).

Frequencies (n) and percentages (%) were used to present the descriptive characteristics of the data while numerical variables were represented through mean ± standard deviation (sd) and median. Kolmogorov-Smirnov test was used for normal distribution analysis. The Mann-Whitney U test and independent-sample t-test were used to compare the two groups in terms of the continuous variables. Categorical data were compared with Chi-Square test or Fisher's Exact test. A p-value <0.05 was considered as statistically significant. The analyses were performed using IBM SPSS-22 (Statistical Package for Social Sciences, Chicago, IL, USA).

## Results

A total of 711 PLWHA were included. Of these, 633 (89.0%) were male and the mean age was 36.53 ± 11.55 years (range, 18–79). The probable route of transmission was heterosexual intercourse in 329 (46.3%) patients and homosexual intercourse in 143 (20.1%) patients. One hundred-ten (15.5%) patients described themselves as bisexual. The CD4+ lymphocyte count <200/mm^3^, 200–350 and ≥350/mm3 were reported in 184 (25.9%), 174 patients (24.6%) and 353 patients (49.6%), respectively. HIV RNA <100,000 and ≥100,000 were reported in 395 patients (55.6%) and 316 patients (44.4%), respectively. Mean CD4+ lymphocyte count at ART initiation was 373.03 ± 240.55 cells/mm3 (range, 0–1450) and mean plasma HIV RNA was 4.88 ± 1.01 log10 iu/mL. Mean duration since ART initiation was 7.54 ± 14.84 months. We found that candidiasis was the most common AIDS-defining opportunistic infection (n=28, 3.9%) followed by, pneumocystis pneumonia (n=16, 2.3%), cytomegalovirus infection (n=6, 0.8%), and cryptococcosis (n=1, 0.1%); while Kaposi's sarcoma (n=11, 1.6%) was the most common AIDS-defining malignancy. There were no significant differences between the included (n=711) and excluded patients (n=95) in terms of age (p=0.379), gender (p=0.094), mean CD4^+^ lymphocyte count (p=0.431) and plasma HIV RNA at ART initiation (p=0.436), and duration since ART initiaion (p=0.453).

Among TB co-infected patients (n=38, 5.3%), there were 34 (89.5%) male. The mean age was 37.89 ±11.45 years (range, 17–79). Median duration since ART initiation was 1 (1–82) month. Mean CD4+ lymphocyte count at ART initiation was 218.55 ± 190.74 cells/mm^3^ (range, 5–839) and mean plasma HIV RNA was 5.11 ± 0.79 log10iu/mL. CD4+ lymphocyte count <200, 200–350 and ≥350 cells/mm3 were reported in 20 (52.6%), 11 patients (28.9%) and 7 patients (18.4%), respectively (p<0.001). HIV RNA <100,000 and ≥100,000 were reported in 15 patients (3.8%) and 23 patients (7.3%), respectively. All active TB patients were prescribed anti-TB treatment. Mean CD4+ lymphocyte count at ART initiation was 373.03 ± 240.55 cells/mm3 (range, 0–1450) and mean plasma HIV RNA was 4.88 ± 1.01 log10 iu/mL. Mean duration since ART initiation was 7.54 ± 14.84 months. Of these, 21 (55.3%) were hospitalized, and two (5.3%) died during anti-TB treatment. Unmasking Immune Reconstitution Inflammatory Syndrome (IRIS) was recorded in two patients (5.3%), while paradoxical IRIS was recorded in five patients (13.2%). Twenty-five patients (65.7%) had pulmonary, 11 (28.9%) patients had extrapulmonary and 2 (5.3%) patients had both pulmonary and extrapulmonary TB. Four patients had the disseminated form of TB. The remaining extrapulmonary sites were the lymph nodes (n=4), pleura (n=3), gastrointestinal tract (n=1), and central nervous system (n=1). Table 1 shows the demographic characteristics of PLWHA. We found that candidiasis was the most common AIDS-defining opportunistic infection (n=28, 3.9%) followed by, pneumocystis pneumonia (n=16, 2.3%), cytomegalovirus infection (n=6, 0.8%), and cryptococcosis (n=1, 0.1%); while Kaposi's sarcoma(n=11, 1.6%) was the most common AIDS-defining malignancy. There were no significant differences between the included (n=711) and excluded patients (n=95) in terms of age (p=0.379), gender (p=0.094), mean CD4+ lymphocyte count (p=0.431) and plasma HIV RNA at ART initiation (p=0.436), and duration since ART initiation (p=0.453)

**Table 1:** The demographic characteristics of people living with HIV/AIDS

	N	%
**Number of patients**	711	100
**Age**		
18–30	259	36.4
31–40	206	29.0
41–50	153	21.5
51–60	70	9.9
>60	23	3.2
**Gender**		
Male	633	89.0
Female	78	11.0
**Sexual orientation**		
Heterosexual	329	46.3
Homosexual	143	20.1
Bisexual	110	15.5
NS	129	18.1
**CD4+ count at ART initiation (cells/mm^3^)**		
CD4 (≥ 350)	353	49.6
CD4 (200–350)	174	24.6
CD4 (< 200)	184	25.9
**HIV RNA level log10 (iu/mL) at ART initiation**		
<100,000	395	55.6
≥100,000	316	44.4
**Duration since ART initiation**		
<3 months	451	63.4
3–6 months	45	6.3
>6 months	160	22.5
**TB co-infected**	38	5.3
Pulmonary	25	65.7
Extrapulmonary	11	28.9
Both	2	5.3
**Substance abuse**		
Smoking	414	58.2
Alcohol	49	6.9
Drug use	66	9.3
Intravenous drug use	10	1.4
**Viral co-infection**		
Hepatits B	38	5.3
Hepatitis C	7	1.0

NS=Non-specified TB=Tuberculosis ART=Anti-retroviral therapy

Table 2 shows predictors and risk factors for the development of TB co-infection in PLWHA. TB development was not associated with age (p=0.392), gender (p=0.928) and duration since ART initiation (p=0.788). But, TB development was associated with low CD4+ lymphocyte count (p<0.001) and high viral load (p=0.040). Additionally, hospitalization was more common in patients with TB co-infection (p<0.001). Although mortality during a 20-year study period was more common in patients with TB co-infection, there was no statistically significant difference between the two groups (p=0.095).

**Table 2:** Predictors and risk factors for development of tuberculosis co-infection in people living with HIV/AIDS

	In total		TB co-infected		TB non-infected		
	n (%)		n(%)		n(%)		p value
**Number of patients**	711(100)		38(5.3)		673(94.7)		
**Age**							0.392‡
Mean ±sd	36.53±11.55		37.89±11.45		36.46±11.56		
Median	34 (17–76)		35 (19–66)		34 (17–76)		
**Gender**							0.928*
Male	633 (89.0)		34 (89.5)		599 (89.0)		
Female	78 (11.0)		4 (10.5)		74 (11.0)		
**HIV RNA log10 at ART initiation (iu/mL)**							
Mean ±sd	4.88±1.01		5.11±0.79		4.87±1.02		0.157¥
Median	4.83 (2.31–8.66)		5.17 (3.39–6.60)		4.81 (2.31–8.66)		
<100,000	395	55.6	15	38.5	380	56.5	0.040*
≥100,000	316	44.4	23	60.5	293	43.5	
**CD4+ T lymphocyte count at ART initiation (cells/mm^3^)**							
Mean ±sd	373.03±240.55		218.55±190.74		381.75±240.23		<0.001‡
Median	350		181.5		361		
≥ 350	353	49.6	7	18.4	346	51.4	<0.001*
200–349	174	24.6	11	28.9	163	24.2	
100–199	80	11.3	8	21.1	72	10.7	
50–99	43	6.0	4	10.5	39	5.8	
<50	61	8.6	8	21.1	53	7.9	
**Duration since ART initiation (months)**							
Mean ±sd	7.54±14.84		10.02±18.68		7.39±14.58		0.831‡
Median	1 (1–108)		1 (1–82)		1 (1–108)		
<3 months	451	63.4	28	73.7	423	62.8	0.788*
3–6 months	45	6.3	2	5.3	43	6.4	
6 months	160	22.5	8	21.0	152	22.6	
Data not available	55	7.7	0	0	55	8.2	
**Substance abuse**							
Smoking	414	58.2	26	68.4	388	57.6	0.190*
Alcohol	49	6.9	7	18.4	42	6.2	0.004*
Drug use	66	9.3	5	13.2	61	9.1	0.397*
Intravenous drug use	10	1.4	1	2.6	9	1.3	0.425†
**Hospitalization**	215	30.2	21	55.3	194	28.8	<0.001*
Mortality during a 20- year period	47	6.6	5	13.2	42	6.2	0.095*

TB=Tuberculosis ART=Anti-retr ovi ral therapy

* Chi-square was used.

† Fisher's exact test was used.

‡ Mann-Whitney U test was used.

¥ Independent sample t-test was used.

TST was recorded in 352 patients (49.5%). Among patients with TST≥5 (n=108), 56 (50.5%) did not receive TB prophylaxis. However, the remaining patients who did not receive prophylaxis had not developed TB. TB developed in 5 patients who were anergic. These five patients had TB diagnosis after ART initiation. Of the 51 patients without TB, 15 had CD4+ <200 cells/mm^3^. A receiver operating characteristic (ROC) analysis showed that the area under the curves (AUC) of CD4+ lymphocyte count as a predictor for TB development in PLWHA were 0.717 (p<0.001). To predict TB diagnosis, the cut-off value of CD4+ lymphocyte count was 250 cells/mm3, with a sensitivity of 68.42% and a specificity of 70.57% (p<0.001) (Figure).

**Figure:**
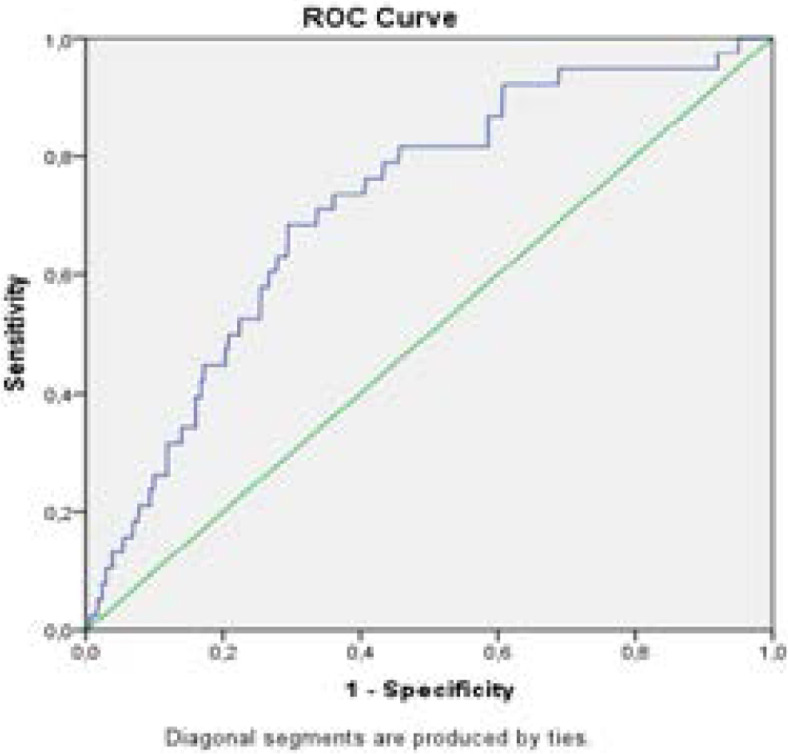
Receiver operating characteristic curve of CD4+ T lymphocyte count for prediction of TB diagnosis in people living with HIV/AIDS, AUC (%95 CI) 0.717(0.638-0.796) p<0.001

## Discussion

Our hospital is one of the most important centers in the country because there are many cases. Also, our study population represents nearly 5.5% of all PLWHA in Turkey. According to the 2019 Global Tuberculosis Report of the World Health Organization (WHO), there were approximately 13,000 (0.016%) PLWHA and 110 (82–140) TB coinfected patients with PLWHA in Turkey by 2018 (https://www.who.int/tb/publications/global_report/en/)^5^. However, the data on TB coinfection in Turkey is limited^6^.

In the present study, the prevalence of TB among PLWHA was 4.5%. According to the WHO, 862,000 out of the 10 million global cases of TB (8.6%) were PLWHA. Better healthcare access and early initiation of ART after 2013 might have contributed to our relatively low TB co-infection frequency^7^–^9^. Also, this may be due to te low prevalence of HIV and low rates of alcohol consumption and substance abuse in Turkey. In our country, screening HIV among TB patients became mandatory in 2011. And as a consequence of this, the incidence of HIV among TB patients increased 5-fold (0.1% vs. 0.5%) in 2016^6^. The Turkish HIV/AIDS Guideline, which is compatible with international guidelines on strategies to screen and therapies to treat HIV/AIDS, has encouraged initiating ART regardless of CD4+ T lymphocyte count since 2013. Additionally, the Social Security Institution's health benefits package in Turkey is comprehensive. Free screning and treatment for TB and HIV are available for all people. Moreover, all patients are mostly screened before surgical procedures. Also, premarital HIV screening is mandatory. However, Turkey is facing a rising threat: the disclosure of risky practices results in social stigma. The social stigma causes those at risk from coming forward for appropriate stewardship of testing, counseling, and treatment^10^. Thus, anonymous HIV screening programs may be beneficial for people who suffer from social stigma.

In the present study, TB development was associated with high viral load (p=0.040). In the study of Fenner et al., they showed that viral load was an important predictor for TB, regardless of CD4+ cell counts7. It may be due to the fact that HIV accelerates the loss of CD4+ T lymphocyte and promotes the progression. In our study, low CD4+ T lymphocyte counts were also associated with a high risk of TB (p<0.001). Of the patients, about a quarter had a CD4+ T lymphocyte count of less than 200 cells/mm^3^ and about a half had more than 350 cells/mm^3^. This finding was consistent with the study of Gumuser et al.^11^. Also, in the study of Lawn et al., CD4+ cell counts were the dominant predictor of TB development during ART8. HIV infection is associated with deterioration of cellular immune responses and increased risk of opportunistic infections via reduced CD4+ T lymphocyte count^11^. In the study of Dravid et al., virologic failure on ART was associated with a higher risk for TB development9. Also, recurrence and relapse are associated with low CD4+ cell counts^1^.

Underlying diseases, immunosuppressive agents, behavioral, social and environmental factors may also play a role as risk factors for TB development^12^–^16^. In a recent study, Miyahara et al. demonstrated that the neutrophil to lymphocyte ratio was a predictor of TB among PLWHA^17^. Substance abuse is one of the most common behavioral risk factor for TB development in PLWHA. In our study, alcohol consumption of more than 40 g of ethanol per day was associated with increased risk of TB in PLWHA. It was consistent with other studies^16^,^18^. In contrast, Rao et al. showed that smoking was an independent risk factor, while alcohol consumption was not^15^.

In our study, two patients had gastrointestinal and miliary TB consecutively, the diagnosis was delayed because the symptoms and signs were subtle. One patient developed pulmonary TB while receiving chemotherapy for laryngeal cancer in the duration of 12 years after ART initiation. Two patients had delayed diagnosis of TB lymphadenitis because TB mimicked the acute retroviral syndrome.

According to the 2018 Official Report of Tuberculosis in Turkey, 61.3% (n=7,616) of all TB cases have pulmonary involvement, 33.6% (n=4,169) have extrapulmonary involvement and 5.1% (n=632) have both the pulmonary and extrapulmonary involvement6. In our study, 71.0%, 23.7% and 5.3% of the patients had pulmonary, extrapulmonary and both pulmonary-extrapulmonary TB respectively, consistent with the overall rates from Turkey.

ART restores and trigger the immune response against opportunistic microorganisms, previously unrecognized tuberculosis can appear within weeks or months after ART initiation. Awareness of TB and TB-related IRIS is crucial in PLWHA. Delayed diagnosis of TB and IRIS whether unmasking or paradoxical may be associated with increased mortality and morbidity, thus an intensive screening latent/active TB before ART initiation is vital to decrease the incidence of TB and IRIS^1^. In our study, unmasking and paradoxical IRIS were recorded in two (5.3%) and five patients (13.2%) respectively but no death occured during TB treatment among these cases. However, due to lack of diagnostic tests and difficulty distinguishing TB-related IRIS from other AIDS-defining opportunistic infections, TB-related IRIS might be underdiagnosed or misdiagnosed^19^ In the study of Bourgarit et al., the rate of TB-related IRIS was 36.8%^20^. In a systematic review and meta-analysis of 13,103 PLWHA, IRIS developed in 15.7% of HIV/TB co-infected patients^21^. In the study, Müller et al. reported that the mortality rate among patients developing TB-related IRIS was 3.5% (n=11).

Mortality among PLWHA and TB is quite high, because of the immunosuppression and opportunistic diseases. A number of studies show that trimethoprim-sulfamethoxazole prophylaxis may be reduce morbidity and mortality in PLWHA with diagnosed TB^22^,^23^. Also, the use of ART significantly reduces mortality rates particularly presence of TB and inpatients with advanced HIV disease^24^. In our study, mortality rate during TB treatment was 5.3%. Some studies have been reported in a variety of settings and the range of mortality which may reach up to 40%^25^–^29^. In the study of Abdullahi et al., 325 of 3163 HIV/TB co-infected patients (10.3%) died within 180 days after initiation of TB treatment^28^. Mabunda et al. found that the mortality rate during a 1-year study period was 12.4%^29^. Gümüşer et al. showed a high mortality rate among their HIV/TB coinfected patients (n=14, 21.2%)9. However they defined the mortality as all cause mortality during a 20-year study period. In our study, all cause mortality during a 20-year study period was 13.2% (n=5).

This study has some limitations. First, it was conducted in a single-center. Second, as a retrospective evaluation was conducted on patient records with data entry prior to the study, missing data could not be completed in some patients. Third, we did not consider underlying comorbid diseases as risk factors and we did not run a multivariate regression analysis. Also, we did not evaluated outcomes, treatment of TB, treatment related adverse events.

This study has also several strengths. First, this study showed a wide data including numerous cases over fifteen years. Second, this study has generalizability because the results are broadly applicable to many different types of people and situations, but still differences in applications of the treatment, prophylaxis and infection control policies between centers may affect the results. In conclusion, HIV and TB co-infection has been a frustrating problem all around the world. There are still clinical challenges to predict TB diagnosis and improve outcomes. However, CD4+ lymphocyte count and viral load may be considered as valuable predictors for TB development in PLWHA. Broadening HIV screening may be the best way to prevent advanced AIDS and curb the TB burden. Also, community strategies to reduce harmful effect of alcohol use should be developed.

## Figures and Tables

**Table 3:** 

CD4+ count Cut-off (cells/mm^3^)	Sensitivity (%)	Specificity (%)
≤ 50	21.1	92.0
≤ 100	31.6	86.2
≤ 200	52.6	75.6
≤ 250	68.4	70.6
≤ 350	81.6	51.3
≤ 500	94.7	26.8
